# Emissions of CO_2_, CH_4_, and N_2_O Fluxes from Forest Soil in Permafrost Region of Daxing’an Mountains, Northeast China

**DOI:** 10.3390/ijerph16162999

**Published:** 2019-08-20

**Authors:** Xiangwen Wu, Shuying Zang, Dalong Ma, Jianhua Ren, Qiang Chen, Xingfeng Dong

**Affiliations:** Heilongjiang Province Key Laboratory of Geographical Environment Monitoring and Spatial Information Service in Cold Regions, Harbin Normal University, Harbin 150025, China

**Keywords:** CO_2_, CH_4_, and N_2_O fluxes, permafrost, forest soil, global warming potential, *Q*_10_

## Abstract

With global warming, the large amount of greenhouse gas emissions released by permafrost degradation is important in the global carbon and nitrogen cycle. To study the feedback effect of greenhouse gases on climate change in permafrost regions, emissions of CO_2_, CH_4_, and N_2_O were continuously measured by using the static chamber-gas chromatograph method, in three forest soil ecosystems (*Larix gmelinii*, *Pinus sylvestris* var. *mongolica*, and *Betula platyphylla*) of the Daxing’an Mountains, northeast China, from May 2016 to April 2018. Their dynamic characteristics, as well as the key environmental affecting factors, were also analyzed. The results showed that the flux variation ranges of CO_2_, CH_4_, and N_2_O were 7.92 ± 1.30~650.93 ± 28.12 mg·m^−2^·h^−1^, −57.71 ± 4.65~32.51 ± 13.03 ug·m^−2^·h^−1^, and −3.87 ± 1.35~31.1 ± 2.92 ug·m^−2^·h^−1^, respectively. The three greenhouse gas fluxes showed significant seasonal variations, and differences in soil CO_2_ and CH_4_ fluxes between different forest types were significant. The calculation fluxes indicated that the permafrost soil of the Daxing’an Mountains may be a potential source of CO_2_ and N_2_O, and a sink of CH_4_. Each greenhouse gas was controlled using different key environmental factors. Based on the analysis of *Q*_10_ values and global warming potential, the obtained results demonstrated that greenhouse gas emissions from forest soil ecosystems in the permafrost region of the Daxing’an Mountains, northeast China, promote the global greenhouse effect.

## 1. Introduction

Global warming has become increasingly prominent. The average surface temperature of the earth has increased by 0.85 °C compared to that before the industrial revolution. This trend is expected to continue and reach an increase of 0.3~4.8 °C by the end of this century [1,2]. The latest analysis of observations from the World Meteorological Organization (WMO) and Global Atmosphere Watch (GAW) program showed that the concentration of CO_2_ was 405.5 ± 0.1 ppm, that of CH_4_ was 1859 ± 2 ppb, and that of N_2_O was 329.9 ± 0.1 ppb in the atmosphere of 2017 [3]. Soil is the main source of greenhouse gas emissions. Almost 5~20% of CO_2_, 15~30% CH_4_, and 60~80% N_2_O are emitted from soils to the atmosphere every year [4], which comprises a key contributor to climate warming. The concentration of greenhouse gas is closely related to the carbon and nitrogen cycles of ecosystems, and its “source-sink” relationship directly affects the response and feedback of ecosystems to climate change [5]. Therefore, the dynamics of three greenhouse gas fluxes have become an important aspect of global climate change research.

Permafrost is the result of energy exchange between the lithosphere and the atmosphere and is also an important part of the cryosphere. Global climate change directly affects both the evolution and development of permafrost. Permafrost soils are large pools of carbon and nitrogen. Jorgenson et al. [6] estimated that one third of the world’s carbon is fixed in soil active layers and permafrost, while 31~102 Pg of total nitrogen is stored in the top 3 m of permafrost [7]. Climate warming will increase the thickness of permafrost active layers, thus releasing the stored ancient carbon and water, and providing more living space and matrix for microorganisms. This increases the emission of greenhouse gases (e.g., CO_2_, CH_4_, and N_2_O), which has a positive feedback effect on the global carbon or nitrogen cycle [8] and climate warming [9]. Therefore, understanding the characteristics of greenhouse gas flux in the permafrost region is of great scientific significance to recognize the carbon and nitrogen cycle of permafrost ecosystems or their response to global climate change.

Forest ecosystems are important players in the global carbon and nitrogen cycle process, and maintain 86% of the plant carbon pool and 73% of the soil carbon pool [10]. With global warming, the forest ecological environment changes, which changes the exchange of greenhouse gases between the atmosphere and forest soils, thus influencing the regional climate. Livesley et al. [11] and Jang et al. [12] found that the canopy, undergrowth litter, roots, and secretions of different tree species differ, resulting in differences in soil physical and chemical properties, microbial community composition and diversity, and changes greenhouse gas emission. Ju et al. [13] found that the soil ecosystem of coniferous forests was highly metabolized, and the CO_2_ flux was significantly higher than that of broadleaf forests. In contrast, Leckie et al. [14] believed that coniferous forest litter was rich in refractory compounds, reducing the soil carbon and nitrogen mineralization rate, and resulting in a lower CO_2_ flux. Wang et al. [15] reported that coniferous forests had thicker litter layer to intercept precipitation, which decreased the soil water content. Coniferous forest soil therefore had a stronger CH_4_ absorption capacity than broadleaf forest soil. Castro et al. [16] believed that the difference in CH_4_ fluxes between both forest types were not significant. The conclusions for soil N_2_O flux in broadleaf forests and coniferous forests were also different. Butterbach-Bahl et al. [17] held that broadleaf forest soil was more likely to form an anaerobic environment and emit more N_2_O. Several scholars have suggested that the N_2_O emission of both forest types are similar, or that coniferous forests are slightly higher [18]. In summary, the effects of different forest types on the flux of CO_2_, CH_4_, and N_2_O in soil remain unclear and need further investigation.

China is one of the world’s three countries with permafrost regions. The permafrost area is about 220 × 10^4^ km^2^ [19]. Northeast China, in which the Xing’an-Baikal permafrost develops (approximately 39 × 10^4^ km^2^), is one of the main distribution areas of permafrost at high latitudes in China, and is to the south of the most prominent part of the Eurasian high-latitude permafrost region [20]. The Daxing’an Mountains area is an important forestry base in China, with a forest coverage rate of 84.32%, which is significantly affected by human activities and climate warming. At present, research on greenhouse gases in Northeast China mainly includes in situ monitoring or indoor simulated incubation experiments, and mostly concentrates on arable land [21,22] or peat swamp wetlands [23,24]. Relatively few studies have addressed greenhouse gas fluxes of forest soils in the permafrost region of the Daxing’an Mountains, and quantitative assessment has not been conducted. This study continuously monitored (from May 2016 to April 2018) the dynamics of greenhouse gas fluxes in different forest types in the permafrost region of the Daxing’an Mountains. The effects of soil temperature, moisture, and other physical and chemical properties on CO_2_, CH_4_, and N_2_O emissions were investigated, and the global warming potential (GWP) and *Q*_10_ were evaluated. The purpose is to assess the greenhouse effect of greenhouse gases, released as a result of permafrost degradation in the Daxing’an Mountains, and provides a basis for the study of carbon and nitrogen budget in the Daxing’an Mountains’ forest ecosystem.

## 2. Materials and Methods

### 2.1. Study Site

**Figure 1:**
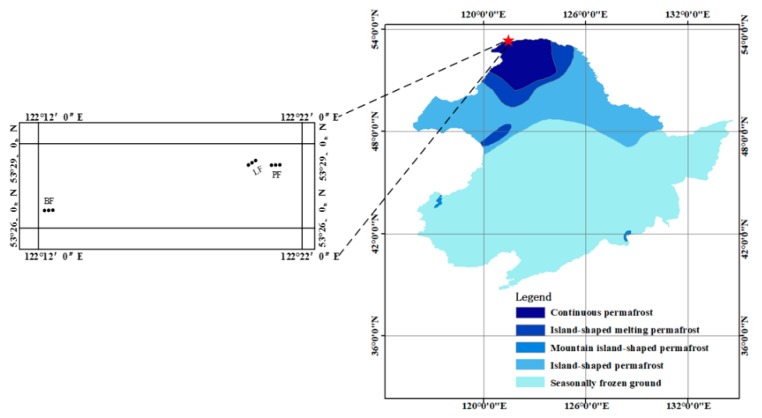
Study region location and sampling design. Black dot represents 5 m × 5 m quadrat.

The study site was situated in the experimental area of the Mohe Forest Ecosystem National Positioning Observation and Research Station in Heilongjiang province (53°17′~53°30′ N, 122°06′~122°27′ E) (Figure 1). The experimental area is located in the permafrost area of the Daxing’an Mountains, with low hills at an average elevation of 300~500 m. It is controlled by a cold temperate continental monsoon climate with an annual average temperature of −3.3 °C and precipitation of 442.95 mm (monitored in 2016 and 2017). In this region, 66.47% of the precipitation is concentrated in summer (from June to August). Furthermore, the soil activity layer freeze-thaw period lasts for about half a year (from mid-April to mid-October), which is slightly longer than the snowpack cover time (from late October to early April). The annual sunshine hours are 2377~2625 h, with an annual total solar radiation of 402~448 kJ cm^−2^, and ≥10 °C annual accumulated temperature of 1436~2062 °C. The zonal soil of the study site is dark brown forest soil. The vegetation in this area belongs to the southern extension of the Eurasian cold-temperate coniferous forest. The zonal vegetation is dominated species of *Larix gmelinii*. Other major species include *Betula platyphylla*, *Pinus sylvestris* var.*mongolica*, *Populus davidiana*, etc. Common plants include *Ledum palustre* var. *dilatatum*, *Rhododendron dauricum*, *Vaccinium uliginosum*, *Vaccinium vitis-idaea*, and *Pyrola incarnate*. Sample selection was based on representative and feasibility principles. After comprehensive investigation, the three most typical forest types in the permafrost region of the Daxing’an Mountains: *Larix gmelinii* forest (LF), *Pinus sylvestris* var.*mongolica* forest (PF), *Betula platyphylla* forest (BF) were selected. One 100 m × 100 m fixed experimental block with similar site conditions was set in each forest type (LF, PF and BF), and a total of 3 experimental blocks were set. All acronyms such as LF, PF, and BF which applicable for all tables and all figures.

### 2.2. Measurement of CO_2_, CH_4_ and, N_2_O Fluxes in Soil

Three 5 m × 5 m quadrats were randomly distributed along the diagonal of each fixed experimental block (100 m × 100 m) in each forest type, but each quadrat was separated by at least 20 m in order to collect greenhouse gas samples. A total of 9 quadrats were set in the three forest types. The static opaque chamber and gas chromatography method were adopted to monitor soil CO_2_, CH_4_, and N_2_O gas fluxes in situ. The static chamber was mainly composed of the chamber and a stainless-steel base. Before sampling, the stainless-steel base with the water groove was buried in the quadrat one week in advance, and was kept fixed during the whole monitoring period to reduce interference by the surrounding environment. The outside of the chamber was affixed with insulation material to decrease the temperature disturbance by the environment inside the box. Three holes were reserved at the top of the chamber to connect the fan power cable, the thermometer probe, and the sampling port, respectively. A 12-V battery-powered fan was installed in the chamber to evenly mix the gas.

In sunny weather, gas samples were collected during mid-morning (9:00~11:00 a.m., local time), which was used to represent one day of average flux [25], and once per week during the growing seasons (from May to September in 2016–2017) and once or twice per month during the non-growing seasons (from October 2016 to April 2017 and from October 2017 to April 2018). The aboveground plant inside the stainless-steel base was cut off 1 day before each sampling during the growing season, while natural snow accumulation was not treated during winter greenhouse gas collection. A medical syringe (60 mL), equipped with a three-way stopcock, was used to collect gas 0, 10, 20, and 30 min after the opaque chamber was sealed with water. When the temperature was below 0 °C, we brought our incubator to store water (300 mL of water per static chamber to water seal), and removed ice or water from the stainless-steel base after each experiment to facilitate the next sampling for water sealing. The samples were immediately transferred to a 200 mL gas sampling bag (Delin gas packing co., Dalian, China) and shipped back to the laboratory for analyzing by 7890B Gas Chromatography (7890B GC System, Agilent, CA, USA).

### 2.3. Soil Sampling and Analysis

In each quadrat (5 m × 5 m), five soil samples (0–15 cm) were randomly collected by removing the surface layer of the soil, and uniformly mixed into one subsample. Each time, 9 soil subsamples were collected from 3 fixed experimental blocks (100 m × 100 m), and total of 216 soil subsamples were collected during the observation period. The atmospheric pressure (BY-2003P barometer, Xieya Electronics Co., Beijing, China), air temperature (Ta), 10 cm soil temperature (T_10_) (Delta TRAK portable thermometer, Delta TRAK, CA, USA) were measured simultaneously with soil sampling. All soil samples were shipped back to the laboratory in ziplock bags for the determination of soil moisture, bulk density (Bd), pH, ammonium nitrogen (NH_4_^+^-N), nitrate nitrogen (NO_3_^−^-N), total nitrogen (TN), and total organic carbon (TOC) (Table 1). Soil moisture was determined by the dry-weighing method. Soil bulk density was determined by the ring-knife method. Soil pH was determined in suspensions composed of 1:5 ratio of air-dried soil and deionized water using a PHSJ-3F pH meter (PHSJ-3F pH, Shanghai, China). Soil NH_4_^+−^-N and NO_3_^−^-N were determined by extraction with potassium chloride solution-spectrophotometric method using a flow injection auto-analyzer (Skalar SAN++, The Netherlands) [26]. Soil TN was determined spectrophotometric using a flow injection auto-analyzer (Skalar SAN++, The Netherlands) [27]. Soil TOC was determined using the dry combustion method by TOC/TN analyzer (Multi C/N 3100, Jena, Germany) [28].

### 2.4. Statistical Analysis

The gas flux was calculated according to the following equation [29]:

(1) $$F=\frac{dc}{dt}\times \frac{M}{V_{0}}\times \frac{P}{P_{0}}\times \frac{T_{0}}{T}\times H$$

where *F* (mg·m^−2^·h^−1^) is the gas flux, *dc*/*dt* is the slope of the linear regression for the gas concentration gradient over time, *M* (g·mol^−1^) is the molecular mass of gas, *P* (Pa) is the atmospheric pressure in sampling site, *T* (k) is the temperature inside the chamber during sampling, *H* (m) is the height of chamber, *V*_0_ (m^3^·mol^−1^), *P*_0_ (Pa) and *T*_0_ (k), are the gas mole volume, atmospheric pressure under standard conditions, and absolute air temperature, respectively. A positive *F*-value means that there is a net emission of gas, and a negative value is the opposite.

The global warming potential (GWP) was calculated according to [30]:

(2) $$\mathrm{GWP}=F_{{\mathrm{CO}}_{2}}+F_{{\mathrm{CH}}_{4}}\times 25+F_{N_{2}O}\times 298$$

where *F*_CO__2_, *F*_CH__4_ and *F*_N__2O_ represent the greenhouse gas emission flux during the monitoring period (t·hm^−2^), 25 and 298 are the conversion factors of CH_4_ and N_2_O, respectively (for a 100 timeframe), to present GWP in t CO_2_ Eq·ha^−1^.

The following equation was established to calculate the temperature sensitivity of gas flux to the changes of T_10_ [31,32]:

(3) $$F=ae^{bT}\;Q_{10}=e^{10b}$$

where *F* is the gas flux, *T* is the soil temperature, coefficient *a* is the intercept of soil respiration when temperature is zero, coefficient *b* represents the temperature sensitivity of soil respiration, *Q*_10_ is the temperature sensitivity.

The differences in soil environment factors among all forests were compared using one-way ANOVA and Tukey’s tests. The relationships of the soil greenhouse gas fluxes with soil temperature and moisture were assessed by regression analysis. The correlations between soil greenhouse gas fluxes and physical and chemical properties were analyzed by Pearson correlation with two tails. SPSS 20.0 (SPSS Inc., Chicago, IL, USA) was used for statistical analysis. All figures were drawn using OriginPro 2016 software (OriginLab Corp., Northampton, MA, USA.).

## 3. Results

### 3.1. The Temporal Variation of Soil CO_2_ Fluxes across LF, PF, and BF

**Figure 2:**
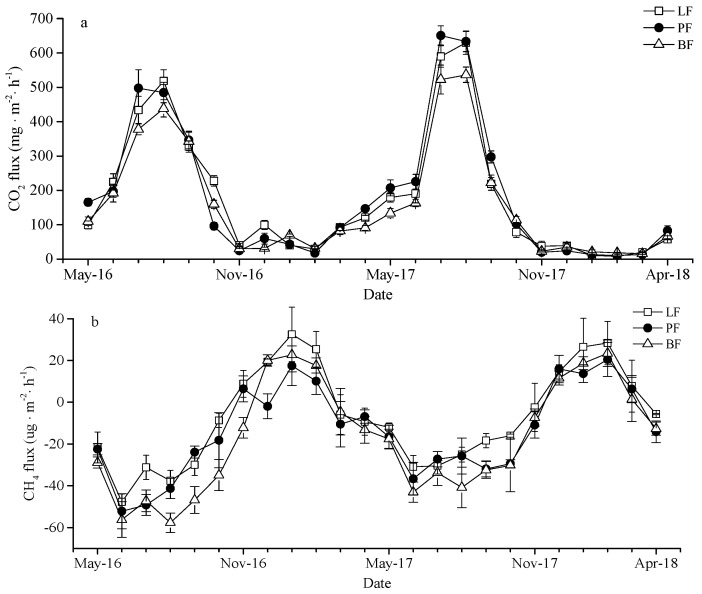

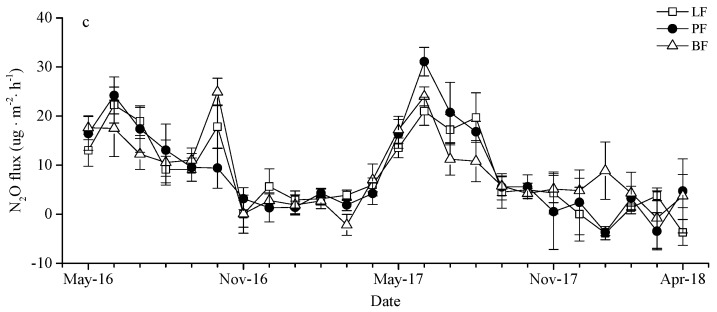
Temporal variation of soil CO_2_ (**a**), CH_4_ (**b**), and N_2_O (**c**) fluxes in LF, PF, and BF.

The temporal variations of soil CO_2_ fluxes in the three forest types in the permafrost region of Daxing’an Mountains were basically exactly the same (Figure 2a). The whole monitoring period was characterized by emissions with significant seasonal variations. The soil CO_2_ fluxes ranged from 7.92 ± 1.30 mg·m^−2^·h^−1^ to 650.93 ± 28.12 mg·m^−2^·h^−1^, and both were unimodal during the two monitoring periods. The majority of emissions were concentrated during the growing season (average fluxes 341.92 ± 202.41 mg·m^−2^·h^−1^), while emissions were relatively low during the non-growing season (average flux 57.92 ± 29.13 mg·m^−2^·h^−1^). PF soil CO_2_ emissions reached a maximum in July (650.93 ± 28.12 mg·m^−2^·h^−1^), and LF and BF reached the maximum emissions in August (629.8 ± 33.91 mg·m^−2^·h^−1^, 536.27 ± 22.36 mg·m^−2^·h^−1^, respectively). PF, LF, and BF reached minimum fluxes of 7.92 ± 1.3 mg·m^−2^·h^−1^ (February), 10.27 ± 3.92 mg·m^−2^·h^−1^ (February), and 15.63 ± 2.31 mg·m^−2^·h^−1^ (March), respectively. The average annual flux of CO_2_ in three forest types followed PF (291.76 mg·m^−2^·h^−1^) > LF (273.84 mg·m^−2^·h^−1^) > BF (243.29 mg·m^−2^·h^−1^). The soil CO_2_ fluxes in coniferous forest were higher than in broad-leaved forest, and PF soil CO_2_ cumulative fluxes were significantly higher than BF (Table 2).

### 3.2. The Temporal Variation of Soil CH_4_ Fluxes across LF, PF, and BF

During the test, the three types of forest soil all showed CH_4_ absorption fluxes (Figure 2b), with apparent seasonal fluctuations. The soil CH_4_ fluxes had similar temporal variation in LF, PF, and BF, which showed increasing fluctuation from June to January of the following year, followed by a gradual decreased until June. The soil CH_4_ fluxes ranged from −57.71 ± 4.65 ug·m^−2^·h^−1^ to 32.51 ± 13.03 ug·m^−2^·h^−1^. During the growing season, all three types of forest soil absorbed CH_4_; however, positive CH_4_ fluxes were found in winter. The highest absorption for CH_4_ fluxes from LF and PF were observed in June (−47.76 ± 3.9 ug·m^−2^·h^−1^, −52.18 ± 8.36 ug·m^−2^·h^−1^), while it was August for BF (−57.71 ± 4.65 ug·m^−2^·h^−1^). The peak fluxes from PF and BF occurred in February (20.43 ± 8.15 ug·m^−2^·h^−1^, 23.46 ± 6.35 ug·m^−2^·h^−1^), and in January for LF (32.51 ± 13.03 ug·m^−2^·h^−1^). The average annual flux of CH_4_ in LF, PF, and BF were −19.33 ug·m^−2^·h^−1^, −24.59 ug·m^−2^·h^−1^, and−30.46 ug·m^−2^·h^−1^, respectively. The soil CH_4_ absorption fluxes in broad-leaved forest were higher than in coniferous forest, and BF soil CH_4_ cumulative absorption fluxes were significantly higher than LF (Table 2).

### 3.3. The Temporal Variation of Soil N_2_O Fluxes across LF, PF, and BF

The soil N_2_O fluxes of the three forest types were largely consistent during the monitoring period of 2016~2018 (Figure 2c). The soil N_2_O fluxes ranged from −3.87 ± 1.35 ug·m^−2^·h^−1^ to 31.1 ± 2.92 ug·m^−2^·h^−1^ in LF, PF, and BF. Emissions during the non-growing season were at a relatively low value with a small amount of absorption and concentrated during the growing season. Soil N_2_O fluxes showed a bimodal trend during 2016–2017, and a unimodal trend during 2017~2018. The maximum soil N_2_O fluxes of LF (22.25 ± 3.66 ug·m^−2^·h^−1^) and PF (31.1 ± 2.92 ug·m^−2^·h^−1^) were recorded in June, and those for BF (24.91 ± 2.8 ug·m^−2^·h^−1^) in October. In contrast, the minimum absorption of N_2_O fluxes were recorded in January (LF: -3.87 ± 1.35 ug·m^−2^·h^−1^, PF: −3.68 ± 0.68 ug·m^−2^·h^−1^) and March (BF: −2.17 ± 2.13 ug·m^−2^·h^−1^), respectively. The average annual flux of N_2_O for PF (13.33 ug·m^−2^·h^−1^) were higher than LF (11.8 ug·m^−2^·h^−1^) and BF (11.45 ug·m^−2^·h^−1^). The soil N_2_O fluxes in coniferous forest were higher than in broad-leaved forest.

### 3.4. Cumulative Soil Greenhouse Gas Emissions and GWP

This study showed that the CO_2_ accumulation fluxes of three forest types in the permafrost region of the Daxing’an Mountains dominated, followed by N_2_O and CH_4_ as absorption fluxes (Table 2). However, LF, PF, and BF showed the “source” of greenhouse gases. The global warming potential is to evaluate the relative impact of greenhouse gases on global climate change using CO_2_ as a reference gas on a 100-year time scale. The radiation effects of CH_4_ and N_2_O were 25 times and 298 times that of CO_2_, respectively [4]. The greenhouse gas GWP value of three forest types showed: PF > LF > BF (Table 2). The study clarified that the release of greenhouse gases from forest soils in the permafrost region of the Daxing’an Mountains has a positive effect on global warming, and the greenhouse gas GWP value of coniferous forests was higher than that of broad-leaved forests.

### 3.5. Effects of Environmental Factors on Soil Greenhouse Gas Fluxes

**Figure 3:**
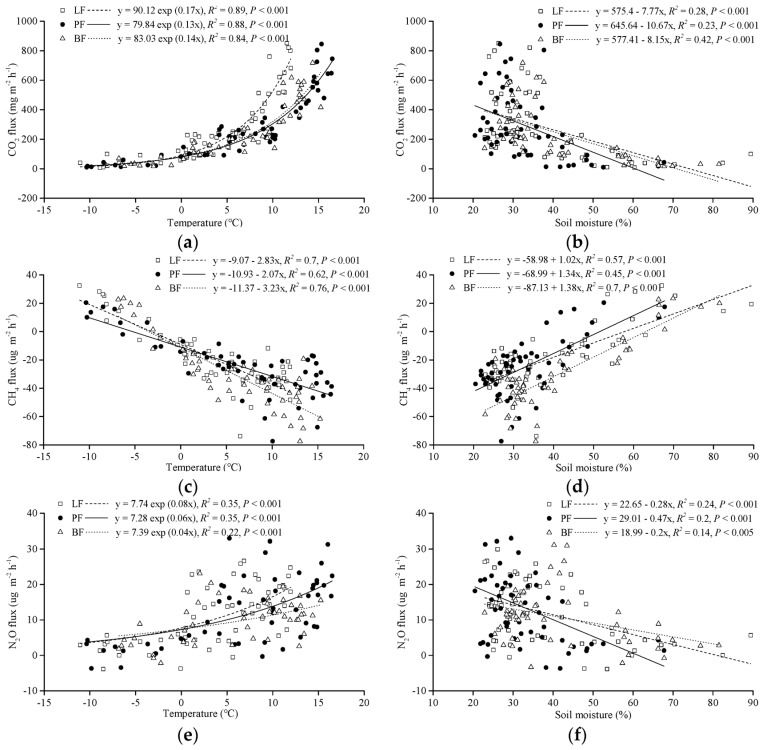
Relationships between (**a**) CO_2_ fluxes and soil temperature, (**b**) CH_4_ fluxes and soil temperature, (**c**) N_2_O fluxes and soil temperature, (**d**) CO_2_ fluxes and soil moisture, (**e**) CH_4_ fluxes and soil moisture, and (**f**) N_2_O fluxes and soil moisture in LF, PF, and BF.

Soil temperature and moisture directly or indirectly affected the production and release of greenhouse gases. The correlations between gas fluxes and soil temperature were higher for CO_2_ (0.84 < *R*^2^ < 0.89, *P* < 0.001; Figure 3a) and CH_4_ (0.62 < *R*^2^ < 0.76, *P* < 0.001; Figure 3b) than for N_2_O (0.22 < *R*^2^ < 0.35, *P* < 0.001; Figure 3c). Soil moisture was positively correlated with CH_4_ fluxes (0.45 < *R*^2^ < 0.7, *P* < 0.001; Figure 3e), while CO_2_ (0.23 < *R*^2^ < 0.42, *P* < 0.001; Figure 3d) and N_2_O (0.14 < *R*^2^ < 0.24, *P* < 0.005; Figure 3f) were negatively correlated. During the study period, the soil CO_2_ and N_2_O fluxes of all forest types showed a significant exponential relationship with T_10_ (*P* < 0.001), the coefficient of variation of the regression model ranged between 0.22 and 0.89. The *Q*_10_ values of CO_2_ fluxes in LF, PF, and BF were 5.47, 3.67, and 4.06, while those for N_2_O were 2.23, 1.82, and 1.49, respectively.

The Person’s correlation analysis between the greenhouse gas fluxes and various environmental factors is showed in Table 3. Three forest-type soil CO_2_ fluxes had a significant negative correlation with TN (*P* < 0.05). The soil CH_4_ fluxes exhibited significant correlation with TOC in LF and BF (*P* < 0.01) and had a significant positive correlation with TN in LF (*P* < 0.05). The soil N_2_O fluxes showed a significant positive correlation with NH_4_^+^-N and NO_3_^−^-N in LF, NO_3_^−^-N in PF (*P* < 0.05), and significant positive correlation with soil pH, NO_3_^−^-N and TN in BF (*P* < 0.01).

## 4. Discussion

### 4.1. Characteristics of Soil CO_2_ Emissions from LF, PF, and BF

This study showed that the seasonal variation of CO_2_ emission fluxes in typical forest LF, PF, and BF in the Daxing’an Mountains were similar, and reached a peak during the growing season (from July to August, Figure 2a), which is consistent with the results of the Song et al. [33] and Li et al. [34]. Soil CO_2_ is mainly derived from autotrophic respiration (mainly vegetation root respiration), heterotrophic respiration (mainly soil microbial respiration), and mineralization decomposition of organic matter. The vegetation in this study area is mostly a shallow root system. During summer, with gradually increasing temperature, the appropriate combination of water and heat in the soil drives root respiration and soil microbial activity, which promotes soil CO_2_ to reach emission peaks. Previous studies reported that the seasonal change of CO_2_ emissions were dominant controlled by soil temperature [32,35]. This study found a consistent result, whereby soil temperature was significantly correlated with CO_2_ fluxes as the dominant environmental variables in affect CO_2_ emissions (Figure 3a). During the non-growing season, soil CO_2_ fluxes fluctuated within a lower range. Analysis of the reasons indicated that root respiration and microbial activity were weak due to low temperature. On the other hand, when the soil was frozen, the active nutrient substrate, which can be directly used by microorganisms was reduced [36], thus resulting in the reduction of soil CO_2_ fluxes. Two years of observations revealed that the temperature in the study area suddenly decreased due to the influence of low environment temperatures at the end of August each year, and the soil CO_2_ fluxes of the three forest types decreased. Then, the soil CO_2_ emission flux value increased, accompanying the increasing temperature back to the normal level of the same period [35]. This study found that the soil CO_2_ fluxes in coniferous forests were higher than that in broad-leaved forests. The PF soil CO_2_ cumulative fluxes were significantly higher than BF. Analysis of the reason found that the PF soil with high temperature had strong microbial metabolic activity, and the humus layer under the forest decomposed faster, releasing a large amount of CO_2_ into the atmosphere. *Q*_10_ is widely used to assess the sensitivity of soil or ecosystem respiration to temperature changes [37]. The *Q*_10_ value of forest soil respiration in China is 1.33~5.53 [38]. The LF soil CO_2_ fluxes (*Q*_10_ = 5.47) are more sensitive than BF (*Q*_10_ = 4.06) and PF (*Q*_10_ = 3.67) to temperature changes, which is consistent with the research results of Zheng et al. [39]. The significant correlation between soil temperature in the study area and soil CO_2_ fluxes indicates strong positive short-term feedback between climate warming and soil CO_2_ fluxes.

### 4.2. Characteristics of Soil CH_4_ Emissions from LF, PF, and BF

CH_4_ is produced by a biogeochemical cycle. It is oxidized by methanotrophs at the soil–water interface or rhizosphere aerobic environment, and the remainder is released into the atmosphere [40]. During this process, methanotrophs and methanogens play a key role. Previous studies have shown that soil CH_4_ as trace gas has both absorption [41,42] and emission [43,44,45]. Soil hydrothermal conditions directly or indirectly change the community characteristics of anaerobic methanogens and aerobic methanotrophs to affect soil CH_4_ fluxes; therefore, the difference in hydrothermal conditions under different ecosystems leads to different soil CH_4_ fluxes [46]. This study observed that the three types of forest soil CH_4_ showed overall absorption. Soil CH_4_ emission, with maximum emission fluxes, only occurred in winter (January or February), and the other seasons showed absorption with the maximum absorption fluxes appearing in June (Figure 2b). After the soil was completely frozen in winter, the soil formed a better anaerobic environment, which was beneficial for the metabolic activity of methanogens and promoted the release of CH_4_. At the beginning of the growing season, the thickness of the permafrost active layer further increases with increasing temperature, which provides a large place for the survival of the methanotrophs [47]. The release of C and N from the frozen microorganisms that were killed in winter provides an important matrix for methanotrophs to accelerate the oxidative absorption of CH_4_ [48]. At the same time, the soil has a short drought period before the rainy season, which is conducive for the spread of atmospheric CH_4_ and O_2_ to the soil and increase the absorption of CH_4_ [49]. During summer, the soil moisture increases due to precipitation, and the soil CH_4_ absorption decreases. The study also found that soil temperature and moisture were significantly related to soil CH_4_ fluxes (Figure 3b,e). Soil CH_4_ average annual absorption flux of three forest types showed BF > PF > LF, and the soil CH_4_ absorption fluxes in the broad-leaved forest were higher than in the coniferous forest, which was consistent with the results of Steudler et al. [50]. The reason for this could be that LF and PF are loamy soils, which are tight and have poor aeration, while the soil bulk density of BF is relatively small, and the gravel content is high. The BF loose soil is conducive to oxygen transport, which enhances the activity of methane oxidase and methane oxidizing microorganisms in soil, which improves the absorption capacity of CH_4_ in BF soil.

### 4.3. Characteristics of Soil N_2_O Emissions from LF, PF, and BF

Soil nitrification and denitrification are two important links in the nitrogen cycle of ecosystems and form an important source of atmospheric N_2_O. The monitoring results showed that N_2_O fluxes of the three forest types were all sources of emissions. In June (during the growing season), soil N_2_O fluxes showed peak emission periods. Thomas et al. [51] also reached a similar conclusion. The main reason may be that the outer surface of the soil particles is covered by an ice layer and the inner layer is wrapped with a tightly bound liquid water film, which forms a better anaerobic environment and remains high active nutrients after the soil completely frozen in winter [52]. The anaerobic environment provides a good place for denitrification to produce N_2_O, which is sequestered by the frozen soil [53]. The N_2_O enclosed in the soil is burst out into the atmosphere after thawing. In summer, plants grow vigorously and absorb a large amount of nitrogen. Competition between vegetation and microbes causes soil microbes to utilize substrate reduction, and frequent rainfall during the summer causes the shallow soil to alternate between wet and dry conditions, all of which affects the soil N_2_O emission rate [54]. Permafrost soils, which are characterized by cold temperatures, have low net N mineralization rates and availability of mineral nitrogen [36]. Consequently, the available nitrogen in the soil (predominantly ammonium nitrogen and nitrate nitrogen) is poor in the forests of northern China [55]. This study found that PF soil N_2_O fluxes were higher than LF and BF. The available nitrogen (NH_4_^+^-N and NO_3_^−^-N) contents of different forest types showed that BF was significantly higher than both PF and LF (*P* < 0.05), and the NH_4_^+^-N content was significantly higher than the NO_3_^−^-N content (*P* < 0. 05) (Table 1). Therefore, denitrification may be the main source of soil N_2_O fluxes in this region. However, BF soil pH was significantly lower than LF and PF (*P* < 0.05) (Table 1). According to Struwe et al. [56], the optimal pH range for denitrification in soil is between 6 and 8. Soil denitrification is inhibited under acidic conditions and the rate of denitrification decreases with increasing soil acidity. Therefore, the N_2_O emission rate of LF was lower than that of PF and BF. Our study found that the temperature sensitivity of N_2_O in LF, PF, and BF were 2.23, 1.82, and 1.49, respectively, and LF soil N_2_O responded most to temperature rise. However, the difference of N_2_O fluxes between the three forest types were not significant, and the forest type was not the main factor affecting soil N_2_O fluxes.

## 5. Conclusions

(1) The typical forest soil of the Daxing’an Mountains permafrost region was a “source” of CO_2_ and N_2_O and a “sink” of CH_4_. The three greenhouse gas fluxes showed strong temporal variety, while the fluxes varied depending on the different sites. The PF soil CO_2_ fluxes were significantly higher than BF. At the same time, the soil absorption fluxes of CH_4_ in BF were significantly higher than that in LF.
(2) Soil temperature and moisture were key environmental factors that correlated the CO_2_ and CH_4_ fluxes of different forest types in this high-latitude permafrost region. *Q*_10_ values showed that LF soil greenhouse gas fluxes were more sensitive to temperature. The N_2_O fluxes were mainly correlated by the soil nitrogen content.
(3) Against the background of climate warming, the CO_2_ and N_2_O emission rates of the three forest types increased with increasing temperature, and the CH_4_ absorption rate decreased, thus enhancing the atmospheric greenhouse effect. On a 100-year time scale, the greenhouse gas GWP of the three forest soil systems in the Daxing’an Mountains permafrost region was positive, which had positive feedback on global warming.

The Daxing’an Mountains permafrost region is extremely sensitive to climate change. The CO_2_, CH_4_, and N_2_O fluxes in the study area had significant emission potential. Temperature alteration leads to complex changes in hydrothermal conditions that either directly or indirectly affect forest ecosystems in the cold region, which transforms ground-gas exchange ratios to affect greenhouse gas fluxes. In the future, stable isotope tracing and microbial high-throughput sequencing technologies will be comprehensively used to analyze the mechanism of soil greenhouse gas accumulation, conversion, and transmission in permafrost region.

## Figures and Tables

**Table 1:** The surface soil (0~15 cm) physicochemical properties in LF, PF, and BF (mean ± SD, *n* = 216).

Blocks	pH	Bd (g·cm^−3^)	NO_3_^−^-N (mg·kg^−1^)	NH_4_^+^-N (mg·kg^−1^)	TN (g·kg^−1^)	TOC (g·kg^−1^)	C/N
LF	5.53 ± 0.23a	1.01± 0.08a	1.57 ± 0.54ab	5.01 ± 0.91c	3.30 ± 0.97ab	52.22 ± 6.21ab	15.82ab
PF	5.57 ± 0.18a	1.05 ± 0.06a	1.31 ± 0.34b	6.67 ± 1.05b	2.91 ± 0.77b	48.36 ± 4.77b	16.62a
BF	4.65 ± 0.17b	0.71 ± 0.09b	2.10 ± 0.42a	10.23 ± 1.24a	4.16 ± 0.85a	58.50 ± 5.17a	14.06b

Note: Lowercase letters indicate differences in physical and chemical indicators between different forest types (*P* < 0.05).

**Table 2:** Average cumulative fluxes and GWP of greenhouse gases from LF, PF, and BF.

Blocks	CO_2_ (t·hm^−2^)	CH_4_ (kg·hm^−2^)	N_2_O (kg·hm^−2^)	GWP (t CO_2_ Eq·hm^−2^)
LF	15.737 ± 1.14ab	−0.639 ± 0.19a	0.715 ± 0.14a	15.934 ± 1.18ab
PF	16.249 ± 0.38a	−1.208 ± 0.28ab	0.757 ± 0.02a	16.445 ± 0.38a
BF	13.876 ± 0.61b	−1.483 ± 0.40b	0.756 ± 0.03a	14.064 ± 0.61b

Note: Lowercase letters indicate differences in indicators between different forest types (*P* < 0.05).

**Table 3:** Relationship between greenhouse gas fluxes and soil physical and chemical properties in LP, PF, and BF.

Blocks	Flux	pH	NO_3_^−^-N (mg·kg^−1^)	NH_4_^+^-N (mg·kg^−1^)	TN (g·kg^−1^)	TOC (g·kg^−1^)
LF	CO_2_	0.235	−0.311	−0.234	−0.459 *	0.163
	CH_4_	0.236	−0.143	−0.318	0.471 *	−0.559 **
	N_2_O	−0.336	0.444 *	0.549 **	−0.104	0.200
PF	CO_2_	−0.095	−0.228	−0.164	−0.506 *	0.411
	CH_4_	0.146	0.127	−0.133	0.202	−0.198
	N_2_O	0.156	0.478 *	0.407	0.202	−0.044
BF	CO_2_	−0.409	−0.477 *	−0.168	−0.509 *	0.160
	CH_4_	0.122	−0.034	0.095	0.246	0.692 **
	N_2_O	0.547 **	0.605 **	0.367	0.570 **	0.105

Note: ** Correlation is significant at the 0.01 level, * correlation is significant at the 0.05 level.
